# Harnessing plant growth-promoting microorganisms to improve drought resilience in common bean (*Phaseolus vulgaris* L.)

**DOI:** 10.1186/s12870-026-09245-8

**Published:** 2026-06-25

**Authors:** Heba I. Mohamed, Abd-alrahman Walid Eid, Kerolos Emad Khalaf, Kirollos Kamal Joseph, Mahmoud Abdulaziz Mohamed, Mahmoud Maged Mohamed, Amira Mohamed Ghanaim

**Affiliations:** Biological and Geological Sciences Department, Faculty of Education, Ain Shams University, Cairo, 11341 Egypt

**Keywords:** Drought stress, Enzymatic antioxidants, *Phaseolus vulgaris*, Photosynthetic pigments, Non-enzymatic antioxidants, Osmolytes, Secondary metabolites

## Abstract

**Background:**

Drought stress is one of the principal abiotic stresses that constrain crops yield across the globe. The current research examines the involvement of PGPM, namely *Bacillus subtilis* (1211 EMCCN) and *Aspergillus niger* (ATCC 102), in improving drought tolerance of Phaseolus vulgaris cultivated in soil water contents of 100%, 60%, and 40% field capacity.

**Results:**

*B. subtilis* showed a higher production of indole-3-acetic acid, cytokinin, and ACC deaminase, indicating its strong role in promoting plant growth and mitigating stress. In contrast, *A. niger* produced more gibberellic acid and enhanced phosphate solubilization. Drought stress significantly reduces plant growth, biomass, chlorophyll content, and relative water content, while increasing electrolyte leakage and oxidative stress markers. Inoculating plants with plant growth-promoting microorganisms (PGPM) alleviated these negative effects, improving growth performance, photosynthetic pigment content, and water status compared to uninoculated plants. The microbial treatments reduced oxidative damage and enhanced antioxidant defense systems, including both enzymatic and non-enzymatic antioxidants. PGPM inoculation also promoted the accumulation of osmolytes and secondary metabolites, aiding osmotic adjustment and redox balance. Multivariate analysis confirmed the positive effects of microbial inoculation in mitigating drought stress.

**Conclusion:**

Among the tested microorganisms, *B. subtilis* showed greater effectiveness than *A. niger* in most of the physiological and biochemical traits measured. Overall, the findings suggest that PGPMs are a promising and sustainable strategy for enhancing drought tolerance and improving the productivity of common beans in water-limited conditions.

**Graphical abstract:**

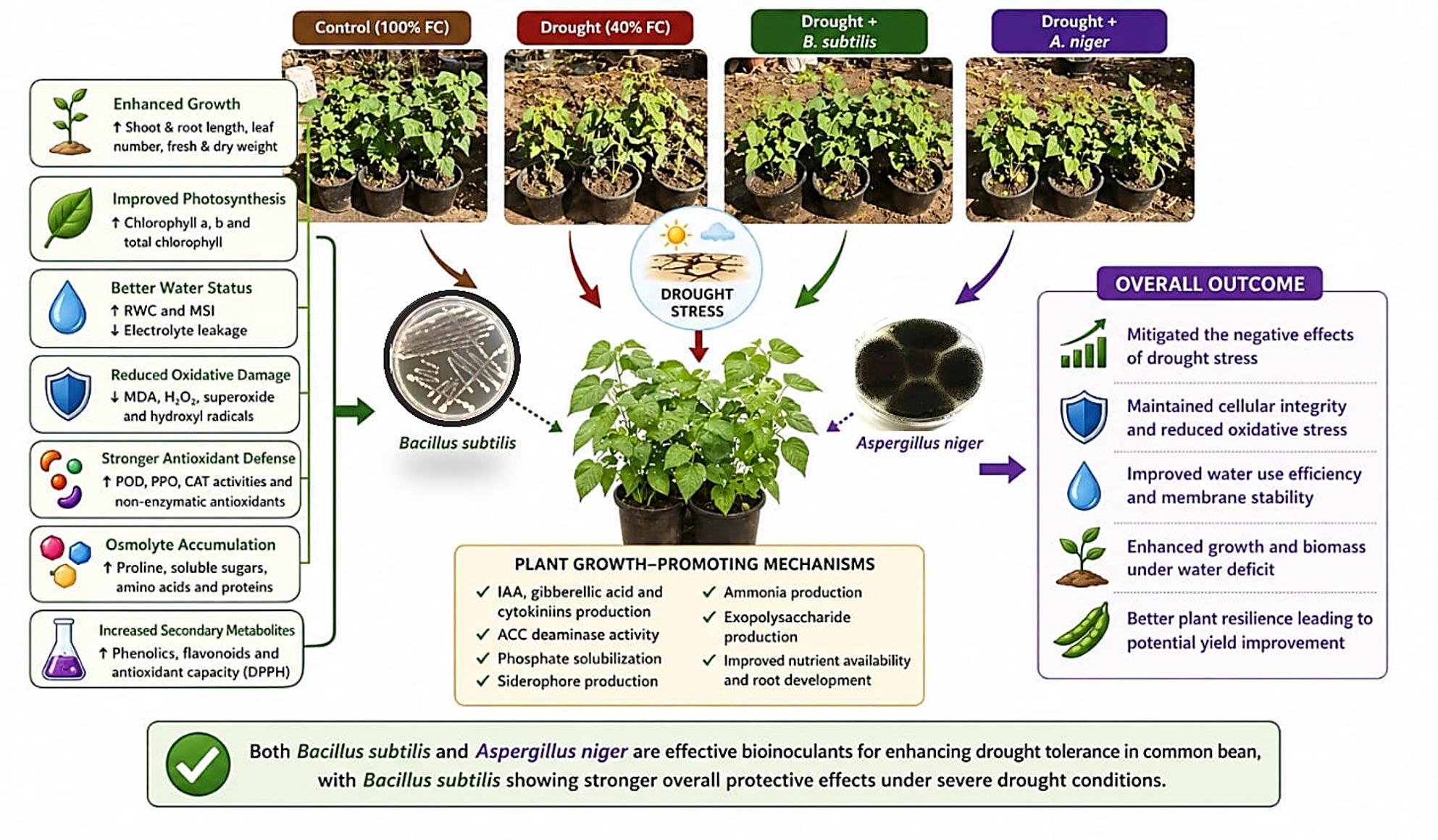

## Introduction

 Climate change has intensified the frequency and severity of drought events worldwide, leading to increased water scarcity and threatening agricultural productivity, particularly in rainfed cropping systems [1]. Irregularities in rainfall patterns, elevated temperatures, and high evapotranspiration rates collectively exacerbate soil moisture deficits, which significantly limit crop growth and yield [2]. Among major abiotic stresses, drought is considered one of the most destructive factors affecting global food security, especially in arid and semi-arid regions where agriculture largely depends on rainfall [1, 3].

Common bean (*Phaseolus vulgaris* L.) is one of the most important grain legumes globally due to its high nutritional value, serving as a rich source of proteins, minerals, and dietary energy [4]. However, it is highly sensitive to water deficit conditions, particularly during critical growth stages such as flowering and pod filling. Drought stress adversely affects physiological and biochemical processes in common bean, including photosynthesis, stomatal regulation, and nutrient uptake, ultimately resulting in reduced biomass accumulation and yield loss [5]. At the cellular level, drought stress induces excessive production of reactive oxygen species (ROS), which causes oxidative damage to lipids, proteins, and nucleic acids, thereby impairing plant growth and development [6].

Conventional breeding approaches and genetic improvement strategies for drought tolerance are often limited by the complex polygenic nature of stress resistance and the long time required for cultivar development. As a result, there is growing interest in sustainable and eco-friendly alternatives, particularly the use of plant growth-promoting microorganisms (PGPMs) [7]. PGPMs include a diverse group of beneficial bacteria and fungi that colonize the rhizosphere or plant tissues and enhance plant growth through multiple direct and indirect mechanisms [8].

Plant growth-promoting rhizobacteria (PGPR) represent one of the most widely studied groups of PGPMs. These bacteria enhance plant drought tolerance by improving root architecture, increasing water and nutrient uptake, regulating phytohormone production, and reducing stress-induced ethylene levels through 1-aminocyclopropane-1-carboxylate (ACC) deaminase activity [9]. In addition, PGPR contributes to osmotic adjustment by promoting the accumulation of compatible solutes such as proline and enhancing antioxidant defense systems, thereby reducing oxidative damage under drought stress [10].

Similarly, plant growth-promoting fungi (PGPF), including species of Aspergillus, have emerged as important bioinoculants for improving plant stress tolerance. *Aspergillus niger* is known to enhance plant growth through phosphate solubilization, organic acid production, siderophore synthesis, and phytohormone modulation [11]. These mechanisms collectively improve nutrient availability, root development, and stress resilience in plants exposed to adverse environmental conditions [12]. Recent studies also suggest that fungal inoculation can improve water-use efficiency and antioxidant activity under drought stress conditions [13].

In common bean, PGPMs have been reported to enhance drought tolerance by improving physiological performance, maintaining relative water content, stabilizing cell membranes, and reducing oxidative stress through enhanced antioxidant enzyme activity [14]. These beneficial effects ultimately translate into improved growth, yield components, and overall plant performance under water-limited conditions [15–18]. Although several studies have examined the role of *B. subtilis* or *(A) niger* individually in drought stress mitigation, comparative investigations integrating both bacterial and fungal plant growth-promoting microorganisms under different drought intensities in common bean remain limited. The novelty of the present study lies in the integrated evaluation of microbial plant growth-promoting traits alongside detailed physiological, biochemical, osmolyte, enzymatic, and non-enzymatic antioxidant responses under graded drought stress conditions. The study also highlights the functional differences between *(B) subtilis* and *A. niger* in drought mitigation mechanisms, providing a more comprehensive understanding of microbial-assisted drought tolerance in *P. vulgaris*.

## Materials and methods

### Plant material and microorganisms

Seeds of common bean (*Phaseolus vulgaris* L. cv. Master B) were kindly provided by the Agricultural Research Center, Ministry of Agriculture, Giza, Egypt. For this study, *Bacillus subtilis* (1211 EMCCN) and *Aspergillus niger* ATCC 102 cultures were provided by the Egyptian Microbial Culture Collection (EMCCN) of the Microbial Resource Center (Cairo, MIRCEN), Faculty of Agriculture, Ain Shams University, Cairo, Egypt.

### Preparation of microbial inoculation

In the case of microbial treatment, bacteria *B. subtilis* and fungus *(A) niger* were cultivated for preparing the microbial inoculum. *(B) subtilis* were grown in nutrient broth medium for 48 h in shaking flask culture at 30 °C and a speed of 150 rpm. After that, the culture was centrifuged at a speed of 8000 rpm for 10 min to obtain a pellet. This pellet was again suspended in sterile distilled water to obtain a 1 × 10⁸ CFU $mL^{-1}$ bacteria solution. The cell-free supernatant obtained after centrifugation was also collected as the bacterial inoculum. On the other hand, the fungus *A. niger* was cultured in potato dextrose broth medium for 7 days without shaking at 28 °C. The fungal culture was separated from the broth medium using Whatman No. 1 filter paper, and the filtrate was collected as the fungal inoculum. The mycelium obtained from the fungal culture was suspended in sterile distilled water to a concentration of 1 × 10⁷ spores $mL^{-1}$. The concentration of the bacterial inoculum (1 × 10⁸ CFU $mL^{-1}$) and that of the fungal spore suspension (1 × 10⁷ spores $mL^{-1}$) were chosen because they are similar to those used in previous studies involving PGPMs that demonstrated successful colonization and increased drought resistance in plants [19–21].

## Determination of plant growth-promoting traits

### Indole-3-Acetic Acid (IAA) production

The determination of IAA levels was performed according to the Salkowski colorimetric technique [22]. Microorganisms were cultured in LB broth medium enriched with 0.5 g $L^{-1}$ of tryptophan at 30 °C for 48 h for bacteria and 28 °C for 72 h for fungi. The medium was centrifuged at 10,000 rpm for 10 min, after which 2 mL was added to 2 mL of Salkowski reagent [1 mL 0.5 M FeCl₃ in 50 mL of 35% perchloric acid solution]. The mixture was kept in the dark for 30 min, and the pink color developed was measured at 530 nm.

### Gibberellic Acid (GA₃) production

GA₃ production was estimated using the method described by Holbrook et al. [23]. Bacterial growth was achieved in Czapek-Dox liquid medium for five days at 28 °C, followed by separation through centrifugation at 10,000 rpm for 10 min. The supernatant obtained was then purified by adding zinc acetate and potassium ferrocyanide solutions, and absorbance was estimated at 254 nm.

### Cytokinin production

Cytokinin production was determined using an adapted colorimetric test method as suggested by Letham [24]. Nutrient broth medium containing yeast extract was incubated for 72 h. Ethyl acetate extraction of the supernatant obtained after centrifugation was followed by evaporation of the solvent, dissolving the residue in methanol, and absorbance readings at 269 nm against kinetin as standards.

### ACC deaminase activity

The ACC deaminase enzyme activity was measured following the protocol described by Viterbo et al. [25]. First, microbial isolates were grown in tryptic soy broth for 24 h, and then they were shifted into a minimal medium containing 3 mM of 1-aminocyclopropane-1-carboxylate (ACC), serving as the only source of nitrogen, and further incubated at 30 °C for 48 h. Bacterial cells were centrifuged, washed, and resuspended in Tris-HCl buffer (pH 8.5). Enzyme activity occurred after incubation of bacterial suspension in the presence of ACC for 30 min. Then, the reaction was stopped, and the quantity of formed α-ketobutyrate was estimated using the DNPH reagent; the absorbance of the obtained complex was measured spectrophotometrically at 540 nm, and ACC deaminase activity was assessed based on a standard curve of α-ketobutyrate.

### Phosphate solubilization

For quantitative determination of phosphate solubilization ability of the tested fungi and bacteria, cultures were grown in Pikovskaya broth, and the cultures were incubated at 28 ± 2 °C for seven days. Following incubation, the filtrate was obtained either by filtration through Whatman No. 1 filter paper or by centrifugation at 8000 rpm for 10 min. The quantity of soluble phosphorus released into the culture media was estimated by the molybdenum blue assay. In brief, a sample of the filtrate was treated with ammonium molybdate reagent and then reduced with a reducing agent. The absorbance of the colored solution was measured spectrophotometrically at 882 nm wavelength, and the quantity of soluble phosphorus was calculated based on a standard curve drawn by using different concentrations of KH₂PO₄ [26].

### Siderophore production

Siderophore synthesis was determined using the Chrome Azurol S (CAS) method as explained by Andrews et al. [27]. Bacterial isolates were cultured on CAS media and grown at 28 to 30 °C for 3 to 5 days. Formation of orange halos signified siderophore synthesis. The quantity was calculated by mixing bacterial growth supernatant with the CAS dye, followed by measuring absorbance at 630 nm.

### Ammonia production

The production of ammonia was done by inoculating the bacteria on peptone water and incubating it at 30 °C for 48 h. Nessler’s reagent was added to check the production of ammonia based on the development of yellow to brown color. The absorbance was read using a spectrophotometer at 450 nm.

### Exopolysaccharide (EPS) production

The production of EPS was evaluated by growing isolates on yeast extract-mannitol broth for 72 h as per the procedure described by Tallon et al. [28]. EPS present in the culture broth was precipitated by adding three volumes of cold ethanol after centrifuging the cultures. The amount of EPS obtained was estimated based on the phenol-sulfuric acid procedure with glucose as standard [29].

### Experimental procedures

Seeds of common bean (*Phaseolus vulgaris* L. cv. Master B) were surface sterilized to minimize microbial contamination. Sterilization was carried out by soaking the seeds in 70% ethanol for 1 min, followed by rinsing thoroughly with sterile distilled water for 3 min. After sterilization, seeds were sown in plastic pots (25 cm in diameter) containing approximately 3.5 kg of sterilized sandy loam soil. The soil had the following characteristics: pH 7.5; electrical conductivity (EC) 1.2 dS $m^{-1}$; cations (meq $L^{-1}$): $Ca^{2+}$ 4.50, $Mg^{2+}$ 1.80, $Na^{+}$ 4.50, $K^{+}$ 0.32; and anions (meq $L^{-1}$): $HCO_{3^{-}}$ 1.35, $SO_{4^{2-}}$ 5.33, Cl⁻ 4.30. Chemical fertilizers (NPK) were applied according to the recommendations of the Egyptian Ministry of Agriculture (kg $ha^{-1}$). The soil under study contained total nitrogen at 0.70 mg $kg^{-1}$, phosphorus at 1.75 mg $kg^{-1}$, and potassium at 17.66 mg $kg^{-1}$. The fertilizers were applied uniformly across all treatments to ensure that nutrient availability was consistent and non-limiting. This approach allowed any observed treatment effects to be primarily attributed to the microbial inoculations applied. Each pot was sown with five seeds, and the experiment was conducted under greenhouse conditions at 25 ± 2 °C, with 60–70% relative humidity and a 14/10 h light/dark photoperiod. In this study, microbial inoculum was prepared in liquid form without the use of carriers. *B. subtilis* was cultured in nutrient broth medium for 48 h at 30 °C and shaking at 150 rpm. On the other hand, *A. niger* was cultivated in potato dextrose broth at 28 °C for 7 days. Thereafter, the cultures were harvested; the bacterial inoculum was adjusted to a concentration of 1 × 10⁸ CFU $mL^{-1}$ while the fungus was adjusted to about 1 × 10⁷ spores $mL^{-1}$. Both inocula were introduced into the soil rhizosphere by soil drenching at planting as well as 7 days after germination of the seeds to facilitate root colonization. In this study, no culture-free fermentation filtrates were used, but only live cultures. For each inoculum treatment, 10 mL of the solution was added directly into the rhizosphere using a sterilized pipette at sowing and again 7 days after seedling emergence to ensure effective colonization. For all pots, there was irrigation up to field capacity until day 14 after sowing. After this period, different treatments were applied to each pot. These treatments are as follows: (1) plants grown with control at 100% FC, (2) plants inoculated with *B. subtilis* at 100% FC, (3) plants inoculated with *(A) niger* at 100% FC, (4) plants exposed to moderate drought (60% FC) without any microbial inoculation, (5) plants inoculated with *(B) subtilis* at 60% FC, (6) plants inoculated with *(A) niger* at 60% FC, (7) plants exposed to severe drought (40% FC) without any microbial inoculation, (8) plants inoculated with *(B) subtilis* at 40% FC, and (9) plants inoculated with *A. niger* at 40% FC. The individual microbial treatments were provided to the plants, but the combined treatment was not included in this study. The soil moisture levels were checked using gravimetric methods, where the pots were weighed every three days and water was added according to the field capacity requirement. This experiment was carried out using a completely randomized design with five replications for each treatment. Plants were collected 45 days after sowing for further analysis.

### Data collection

Forty-five days after sowing, each treatment consisted of five replicates (*n* = 5), with each replicate represented by a plastic pot containing five seeds. Once germination occurred, consistent agronomic practices were applied. For morphological observations, ten plants were randomly selected from each treatment across the replicates, while three plants per treatment were utilized for biochemical analyses. To ensure accurate statistical evaluation and avoid pseudo-replication, the mean values from each replicate (on a pot basis) were used as the experimental unit.

### Biochemical measurements

### Determination of photosynthetic pigments

The contents of chlorophyll a, chlorophyll b, total chlorophyll content, and carotenoids were determined according to the procedures described by Wellburn [30] and Lichtenthaler [31]. Leaf samples (0.5 g) were ground with 80% acetone solution, and the resulting extract was filtered at 5000 × g for 10 min. Absorbance of filtrate was determined at 663 nm (Chl a), 645 nm (Chl b), and 480 nm (carotenoids) using a UV-Vis spectrophotometer. The pigment concentrations were calculated using the following equations:

$$Chl\,a\left(mg\,g^{-1}FW\right)=\left[12.7\times\,A663-2.69\times\,A645\right]\times\,V/\left(1000\times\,W\right)$$

$$Chl\,b\left(mg\,g^{-1}FW\right)=\left[22.9\times\,A645-4.68\times\,A663\right]\times\,V/\left(1000\times\,W\right)$$

$$Total\,Chl\left(mg\,g^{-1}FW\right)=\left[20.2\times\,A663+8.02\times\,A645\right]\times\,V/\left(1000\times\,W\right)$$

$$Carotenoids\left(mg\,g^{-1}FW\right)=\left[A480+\left(0.114\times\,A663\right)-\left(0.638\times\,A645\right)\right]\times\,V/\left(1000\times\,W\right)$$

Where:

- A = absorbance at specific wavelengths
- V = final volume of extract (mL)
- W = fresh weight of sample (g)

### Determination of Relative Water Content (RWC)

RWC was measured using Barrs and Weatherley [32] method. Fresh weight (FW) was obtained by weighing leaf discs of ~ 1 cm². Turgid weight (TW) was obtained by floating the leaf discs in distilled water for 4 h. Dry weight (DW) was obtained by oven-drying the leaves at 70 °C for 48 h. The formula for calculating RWC is:

$$RWC\left(\%\right)=\frac{FW-DW}{TW-DW}\times\,100$$

### Electrolyte Leakage (EL)

The leaves were obtained and made into uniform leaf disks (0.5 to 1 cm²). The leaf disks were then rinsed with distilled water and submerged in test tubes filled with 10 mL of distilled water. They were allowed to be shaken for 2 to 4 h, and the electrical conductivity of the distilled water was then measured using a conductivity meter (C₁). The leaf disks were heated for 10 to 15 min until there was complete cell membrane breakdown. The total electrical conductivity after the boiling process was then measured (C₂). The electrolyte leakage was calculated using the following equation from Lutts et al. [33]:

$$EL\left(\%\right)=\frac{C_{1}}{C_{2}}\times100$$

### Determination of Membrane Stability Index (MSI)

Leaf tissues (0.1–0.2 g) were subjected to an incubation period in distilled water solution (10 mL) at 40 °C for 30 min, after which conductivity was determined (C₁). Leaf tissues were boiled at 100 °C for 10 min, and conductivity was taken (C₂). MSI was obtained using the formula described by Sairam et al. [34].

$$MSI\left(\%\right)=\left[1{-}\frac{{C}_{1}}{{C}_{2}}\right]\times\:100$$

### Determination of lipid peroxidation

To prepare the sample, grind 0.2 to 0.5 g of fresh plant tissue in 5 to 10 mL of cold 0.1% (w/v) trichloroacetic acid (TCA) using a mortar and pestle. Centrifuge the homogenate at 10,000 to 12,000 rpm for 10 to 15 min at 4 °C and collect the supernatant. Add 1 mL of the supernatant to 4 mL of 0.5% (w/v) thiobarbituric acid in 20% TCA and boil for 25 to 30 min at 95 °C. Cool the mixture quickly in ice water and centrifuge at 10,000 rpm for 10 min to eliminate turbidity. Measure the absorbance of the supernatant at 532 nm, accounting for turbidity at 600 nm, and calculate the malondialdehyde concentration using an extinction coefficient of 155 $mM^{-1} cm^{-1}$ [35].

### Determination of hydrogen peroxide

The assay is performed using 0.5 mL of the supernatant solution, which is added to 0.5 mL of 10 mM potassium phosphate buffer (pH 7.0) along with 1 mL of 1 M potassium iodide (KI). This solution is kept in the dark for 10 to 15 min to allow complete development of color. The absorbance is recorded on a spectrophotometer using a 390 nm wavelength. Concentration of hydrogen peroxide can be determined with reference to a standard curve constructed using different concentrations of hydrogen peroxide [36].

### Determination of superoxide radical

The superoxide anion concentration was measured by the nitroblue tetrazolium (NBT) reduction technique. Fresh leaves (0.5 g) were homogenized in 50 mM phosphate buffer (pH 7.8) and centrifuged for 15 min at 12,000 rpm in a refrigerated condition (4 °C). The extract was prepared and used for further measurements. The reaction mixture contained 0.5 mL of plant extract, 0.5 mL of 50 mM phosphate buffer (pH 7.8), and 0.5 mL of 0.05% NBT solution. This mixture was incubated for 30 min at 25 °C in dark. After that, the optical density was recorded at 560 nm [37].

### Determination of hydroxyl radical

The freshly harvested plant tissues (0.2–0.5 g) are homogenized in 3–5 mL of cold 50 mM phosphate buffer (pH 7.0), followed by centrifugation at 10,000–12,000 rpm for 10–15 min at 4 °C. Supernatant fluid is used for further analysis. In the reaction mixture containing 0.5 mL of plant extract, 0.5 mL of 50 mM phosphate buffer (pH 7.0), 0.5 mL of 10 mM salicylic acid, 0.2 mL of 10 mM FeSO₄, and 0.2 mL of 10 mM H₂O₂, formation of hydroxyl radicals will take place. The reaction mixture is kept at 37 °C for 30–60 min. Then the reaction is stopped, and the colorimetric measurement is performed at 510 nm. Color depth depends on the concentration of hydroxylated salicylic acid produced by hydroxyl radicals [38].

### Determination of total phenolic content

The total amount of phenolics was quantified according to the protocol of Rebaya et al. [39] as the Folin-Ciocalteu method. In brief, a sample volume of 0.5 mL was mixed with Folin-Ciocalteu reagent in an amount of 2.5 mL and then 2 mL of Na₂CO₃ solution. Then, it was incubated at room temperature for 30 min, and the absorbance at 765 nm was measured via the spectrophotometer. Total phenolic compounds were calculated using a calibration graph of gallic acid and given in $mg\,GAE\,g^{-1}\,FW$.

### Determination of total flavonoid content

The total amount of flavonoids was measured through the aluminum chloride colorimetric technique, following Zhishen et al. [40]. This was achieved through incubation of the sample (consisting of plant extract prepared through dissolving 0.5 g of plant extract in 80% methanol) together with aluminum chloride and potassium acetate. Absorbance was measured after 30 min at room temperature against 415 nm. Flavonoids were quantified through a quercetin standard calibration curve, expressed in milligrams of quercetin per gram dry weight $\left(mg\,QE\,g^{-1}FW\right)$.

### Determination of antioxidant activity

Determination of antioxidant activity was done by using DPPH free radical scavenging method [41]. This was performed by adding 1 mL of plant extract (obtained from the dissolution of 0.5 g of plant material in 80% methanol) to 2 mL of 0.1 mM DPPH. After 30 min of incubation in darkness at room temperature, the absorbance of the mixture was read at 517 nm. DPPH radical scavenging activity was expressed as percentage inhibition (%).

### Determination of proline

The amount of proline was determined according to the procedure proposed by Bates et al. [42]. A sample of fresh plant material (0.5 g) was ground with 10 mL of 3% sulfosalicylic acid, and the resulting suspension was centrifuged at 10,000 rpm for 10 min. A volume of 2 mL of the supernatant was added to 2 mL of acid ninhydrin and 2 mL of glacial acetic acid, and the mixture was heated to 100 °C for 1 h. After cooling in an ice bath, the mixture was extracted with 4 mL of toluene. The absorbance of the colored layer was read at 520 nm.

### Determination of soluble sugars

The determination of soluble sugar concentration was done using the method of phenol-sulfuric acid [29]. The sample plant (0.5 g) was homogenized in 10 mL of 80% ethanol and then centrifuged at 10,000 rpm for 10 min. One mL of the supernatant liquid was then mixed with 1 mL of 5% phenol and 5 mL of concentrated sulfuric acid. Following an incubation period of 30 min at room temperature, the absorbance was read at 490 nm.

### Determination of total free amino acids

The determination of total free amino acids was performed using the ninhydrin procedure [43]. Plant material (0.5 g) was extracted in 10 mL of 80% ethanol and centrifuged at 10,000 rpm for 10 min. A 1-mL volume of the extract was added to 1 mL of ninhydrin solution and heated at 100 °C for 15 min. The absorbance was read at 570 nm, and the results were expressed as $mg\,g^{-1}\,FW$.

### Determination of total soluble protein

The total amount of soluble protein was measured through the Bradford protein assay [44]. Fresh tissue (0.5 g) was ground with 10 mL of 50 mM phosphate buffer (pH 7.0) and centrifuged at 12,000 rpm for 15 min at 4 °C. One mL of the supernatant was combined with 5 mL of Bradford reagent and incubated at room temperature for 10 min before reading the absorbance at 595 nm. Protein concentration was determined using the Bovine Serum Albumin (BSA) calibration curve, and results were presented in $mg\,g^{-1}\,FW$.

### Determination of antioxidant activity

The activity of the peroxidase enzyme (POD, EC 1.11.1.7) was measured using the method from Chance and Maehly [45]. Fresh plant tissue (0.5 g) was ground in 50 mM phosphate buffer (pH 7.0) and centrifuged at 12,000 rpm for 15 min at 4 °C. For the enzyme reaction, 1 mL of the extract was mixed with 1 mL of 0.05 M pyrogallol and 0.5 mL of 1% hydrogen peroxide, with the absorbance at 420 nm recorded over 3 min. Activity was expressed in units per mg of protein (U $mg^{-1}$ protein). Polyphenol oxidase (PPO, EC 1.10.3.2) activity was evaluated by mixing the enzyme solution with 2 mL of 0.1 M catechol in phosphate buffer (pH 7.0) and recording the absorbance at 410 nm for 3 min [46]. Catalase (CAT) activity was assessed by monitoring the decomposition of hydrogen peroxide (H₂O₂) at 240 nm. The reaction consisted of 1 mL of enzyme solution and 2 mL of 50 mM H₂O₂ in phosphate buffer (pH 7.0), with absorbance changes tracked over 3 min, reported as U $mg^{-1}$ protein [45].

### Determination of ascorbic acid (vitamin C)

The content of ascorbic acid was determined by the method described by Mukherjee and Choudhuri [47]. 0.5 g of freshly collected plant material was extracted in 5 mL of 6% trichloroacetic acid (TCA) and centrifuged at 12,000 rpm for 10 min at 4 °C. 1 mL of the extract obtained after centrifugation was added to 2 mL of 0.4% 2,6-dichlorophenolindophenol (DCPIP) solution, and its absorbance was measured at 520 nm wavelength.

### Determination of tocopherol (vitamin E)

The content of tocopherols was estimated using the procedure described by Quaife and Dju [48]. Plant tissue (0.5 g) was extracted in a mixture of 5 ml ethanol, 1% pyrogallol, and 1 ml of 60% KOH solution. Incubation of this solution at 70 °C for 30 min and subsequent extraction of the petroleum ether fraction were followed by determination of absorbance at 470 nm.

### Determination of glutathione (GSH)

The glutathione concentration was determined through the Ellman reagent method, as explained by Salbitani et al. [49]. In a mortar, 0.5 g of plant sample were crushed using 5 ml of 5% sulfosalicylic acid and then centrifuged at 12,000 revolutions per minute for 10 min. One milliliter of supernatant was combined with 2 ml of DTNB solution at 0.3 mM concentration in phosphate buffer solution at pH 7.0. Absorbance was then recorded at 412 nm, and GSH concentration was quantified in units of $\mu\,M\,g^{-1}\,FW$.

### Determination of anthocyanin

Plant tissue (0.5 g) was ground with 10 mL methanol that contained 1% HCl overnight at 4 °C. It was then centrifuged at 10,000 rpm for 10 min. The absorbance was recorded at 530 and 657 nm. Anthocyanin was determined by using the equation proposed by Giusti and Wrolstad [50].

$$Anthocyanin\left(\mu\,M\,g^{-1}FW\right)=\left(A_{530}- 0.25\times\,A_{657}\right)\times\,V/W$$

Where A₅₃₀ and A₆₅₇ are absorbances at 530 and 657 nm, respectively; V = volume of extract (mL); W = fresh weight of tissue (g).

### Data analysis

The analysis of variance (ANOVA) method was used to study the impact of varying drought conditions and microbial inoculation on the plants. The means were compared using the Tukey’s HSD test, with significance at *p* < 0.05. For generating heatmaps, the web-based application ClustVis, which allows clustering of multidimensional datasets (http://biit.cs.ut.ee/clustvis/), was used [51].

## Results

### Characterization for plant growth–promoting traits

The plant growth-promoting traits of *B. subtilis* and *(A) niger* showed notable differences between the two microorganisms (Table 1). *(B) subtilis* exhibited a higher production of indole-3-acetic acid (IAA), measuring 28.6 ± 1.3 µg $mL^{-1}$, compared to *(A) niger*, which produced 21.4 ± 1.1 µg $mL^{-1}$. Similarly, the production of cytokinins was greater in *(B) subtilis* at 6.5 ± 0.4 µg $mL^{-1}$, while *A. niger* produced 4.7 ± 0.3 µg $mL^{-1}$. In contrast, *(A) niger* demonstrated a higher production of gibberellic acid (GA₃), with a concentration of 15.2 ± 0.9 µg $mL^{-1}$, compared to *(B) subtilis*, which had 12.8 ± 0.7 µg $mL^{-1}$. Phosphate solubilization was also significantly higher in *(A) niger*, at 312 ± 10 µg P $mL^{-1}$, than in *(B) subtilis*, which measured 245 ± 8 µg P $mL^{-1}$. Regarding stress-related traits, ACC deaminase activity was markedly higher in *B. subtilis* at 0.38 ± 0.02 µmol α-ketobutyrate $mg^{-1}\,h^{-1}$, compared to *(A) niger*, which had 0.21 ± 0.01 µmol α-ketobutyrate $mg^{-1}\,h^{-1}$. Additionally, both siderophore and ammonia production were greater in *(B) subtilis*, recording 68.4 ± 3.1% and 19.6 ± 0.9 µg $mL^{-1}$, respectively, while *A. niger* showed lower values of 54.7 ± 2.8% and 16.3 ± 0.8 µg $mL^{-1}$.

**Table 1:** Characterization for plant growth–promoting traits

Trait	Bacillus subtilis	Aspergillus niger
Indole-3-acetic acid (IAA) µg $mL^{-1}$	28.6 ± 1.3	21.4 ± 1.1
Gibberellic acid (GA₃) µg $mL^{-1}$	12.8 ± 0.7	15.2 ± 0.9
Cytokinin µg $mL^{-1}$	6.5 ± 0.4	4.7 ± 0.3
ACC deaminase activity µmol α-ketobutyrate $mg^{-1}\,h^{-1}$	0.38 ± 0.02	0.21 ± 0.01
Phosphate solubilization µg P $mL^{-1}$	245 ± 8	312 ± 10
Siderophore production %	68.4 ± 3.1	54.7 ± 2.8
Ammonia production µg $mL^{-1}$	19.6 ± 0.9	16.3 ± 0.8
Exopolysaccharides (EPS) µg $mL^{-1}$	128 ± 5	142 ± 6

The values represent the means of three replicates with standard deviation (± SD)

### Effect of PGPM on morphological parameters under drought stress

**Fig. 1:**
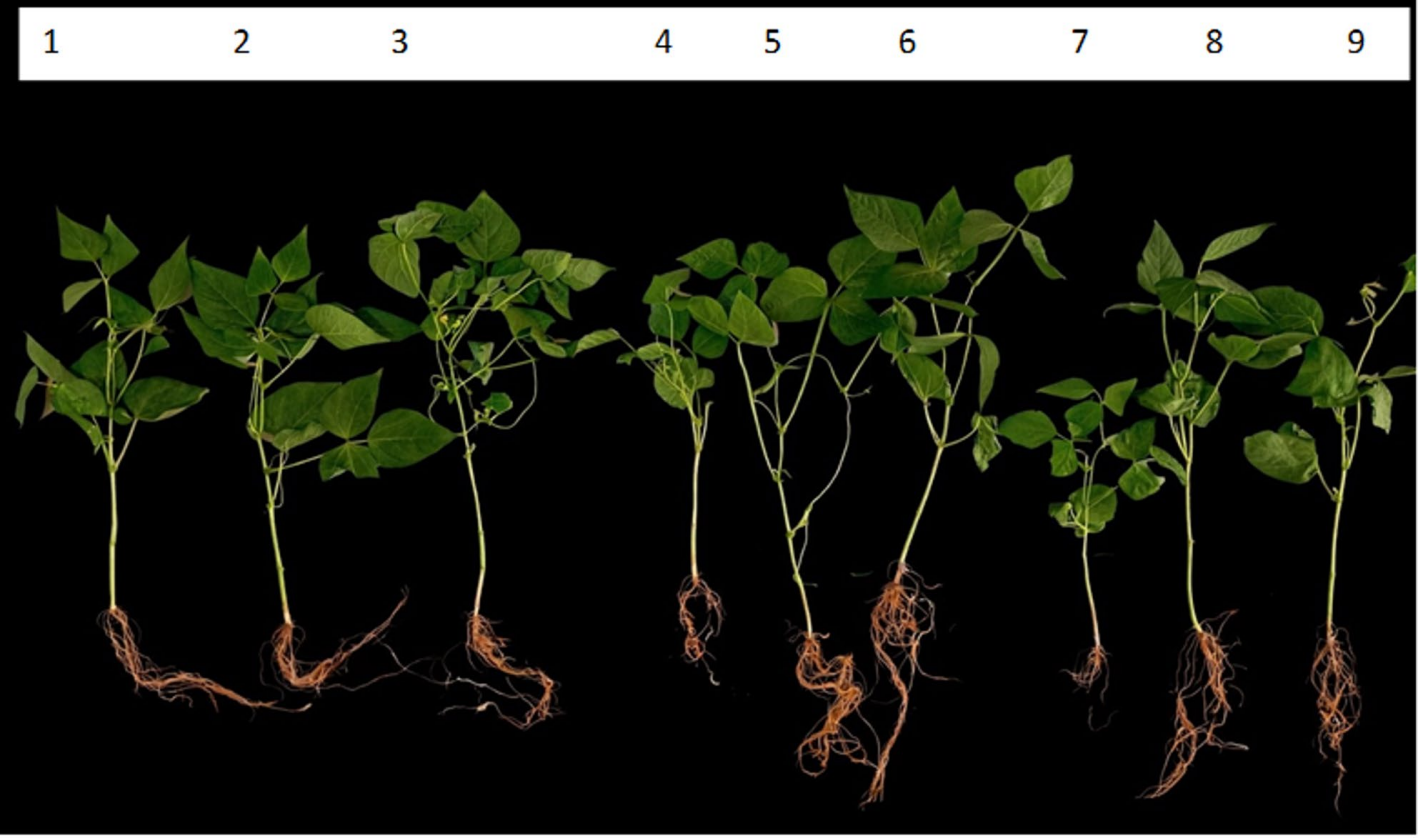
Effect of B. subtilis and A. niger on morphological parameters of common bean plants under drought stress. 1: Control (100% FC), 2: B. subtilis (100% FC), 3: A. niger (100% FC), 4: Drought stress (60% FC), 5: B. subtilis (60% FC), 6: A. niger (60% FC), 7: Drought stress (40% FC), 8: B. subtilis (40% FC), 9: A. niger (40% FC)

Drought stress greatly affected the growth and biomass accumulation of common beans; the more the stress was intense (40% FC), the lower the biomass produced (Fig. 1). However, the application of *B. subtilis* and *(A) niger* enhanced the growth characteristics of common beans, even during water stress. In 100% FC, (B) subtilis had the highest impact on the growth characteristics of common beans, where they enhanced shoot length (45.0 cm), root length (25.0 cm), number of leaves (18), and shoot fresh weight (22.0 g). Under 60% FC, drought stress led to a decrease in shoot length, root length, leaf number, and biomass when compared to the well-watered plants. The application of PGPMs helped in minimizing these adverse effects; however, *B. subtilis* performed better than *(A) niger*. Shoot and root lengths recorded in plants inoculated with *(B) subtilis* were 38.0 cm and 22.0 cm, respectively, while those of *A. niger* were 35.5 cm and 21.0 cm, respectively (Table 2).

**Table 2:** Effect of *B. subtilis* and *A. niger* on morphological parameters on common bean plants under drought stress

Treatment	Shoot Length (cm)	Root Length (cm)	No. of Leaves	No. of Branches	FW of Shoot (g)	DW of Shoot (g)	FW of Root (g)	DW of Root (g)
Control (100% FC)	38.5 ± 1.5^c^	20.0 ± 1.1^e^	14 ± 0.5^d^	4 ± 0.5^c^	18.5 ± 1.0^c^	3.8 ± 0.2^b^	6.5 ± 0.4^c^	1.5 ± 0.1^cd^
*B. subtilis* (100% FC)	45.0 ± 1.8^a^	25.0 ± 1.3^a^	18 ± 0.6^a^	6 ± 0.6^a^	22.0 ± 1.2^a^	4.6 ± 0.3^a^	8.2 ± 0.5^a^	2.0 ± 0.1^a^
*A. niger* (100% FC)	42.0 ± 1.6^b^	23.0 ± 1.2^b^	16 ± 0.7^b^	5 ± 0.5^b^	20.5 ± 1.1^b^	4.3 ± 0.2^a^	7.5 ± 0.4^b^	1.8 ± 0.1^b^
Drought stress (60% FC)	30.2 ± 1.2^e^	18.5 ± 1.0^f^	11 ± 0.4^f^	3 ± 0.4^d^	13.2 ± 0.8^e^	2.6 ± 0.2^d^	4.8 ± 0.3^e^	1.1 ± 0.1^f^
*B. subtilis* (60% FC)	38.0 ± 1.5^c^	22.0 ± 1.2^c^	15 ± 0.5^c^	5 ± 0.5^b^	17.5 ± 0.9^c^	3.5 ± 0.2^bc^	6.5 ± 0.4^c^	1.6 ± 0.1^c^
*A. niger* (60% FC)	35.5 ± 1.4^d^	21.0 ± 1.1^d^	14 ± 0.4^d^	4 ± 0.5^c^	16.0 ± 0.8^d^	3.2 ± 0.2^c^	6.0 ± 0.3^d^	1.4 ± 0.1^d^
Drought stress (40% FC)	22.0 ± 1.0^g^	15.0 ± 0.8^h^	8 ± 0.3^g^	2 ± 0.3^e^	9.0 ± 0.6^g^	1.8 ± 0.1^e^	3.2 ± 0.2^g^	0.7 ± 0.05^g^
*B. subtilis* (40% FC)	30.5 ± 1.3^e^	18.5 ± 1.0^f^	12 ± 0.8^e^	4 ± 0.4^c^	13.0 ± 0.7^e^	2.7 ± 0.2^d^	4.7 ± 0.3^e^	1.2 ± 0.1^e^
*A. niger* (40% FC)	28.0 ± 1.2^f^	17.5 ± 0.9^g^	11 ± 0.7^f^	3 ± 0.4^d^	12.0 ± 0.6^f^	2.4 ± 0.2^d^	4.2 ± 0.2^f^	1.0 ± 0.1^f^

The values represent the means of ten replicates with standard deviation (± SD). Mean values in each column followed by a different lowercase letter are significantly different according to Tukey’s HSD test at *p* ≤ 0.05

The severe drought stress (40% FC) had the most significant impact on plant growth and biomass. In uninoculated plants, the minimum shoot and root lengths were 22.0 cm and 15.0 cm, respectively. Moreover, the minimum fresh weight was recorded at 9.0 g. However, the inoculation of PGPMs was beneficial for enhancing these parameters. Shoot and root lengths increased to 30.5 cm and 18.5 cm, respectively, after *B. subtilis* treatment. After treatment with *(A) niger*, shoot and root lengths increased to 28.0 cm and 17.5 cm, respectively (Table 2). Overall, *(B) subtilis* was more effective than *A. niger* in improving growth and biomass under both normal and drought-stress conditions.

### Effect of PGPM on chlorophyll content under drought stress

**Fig. 2:**
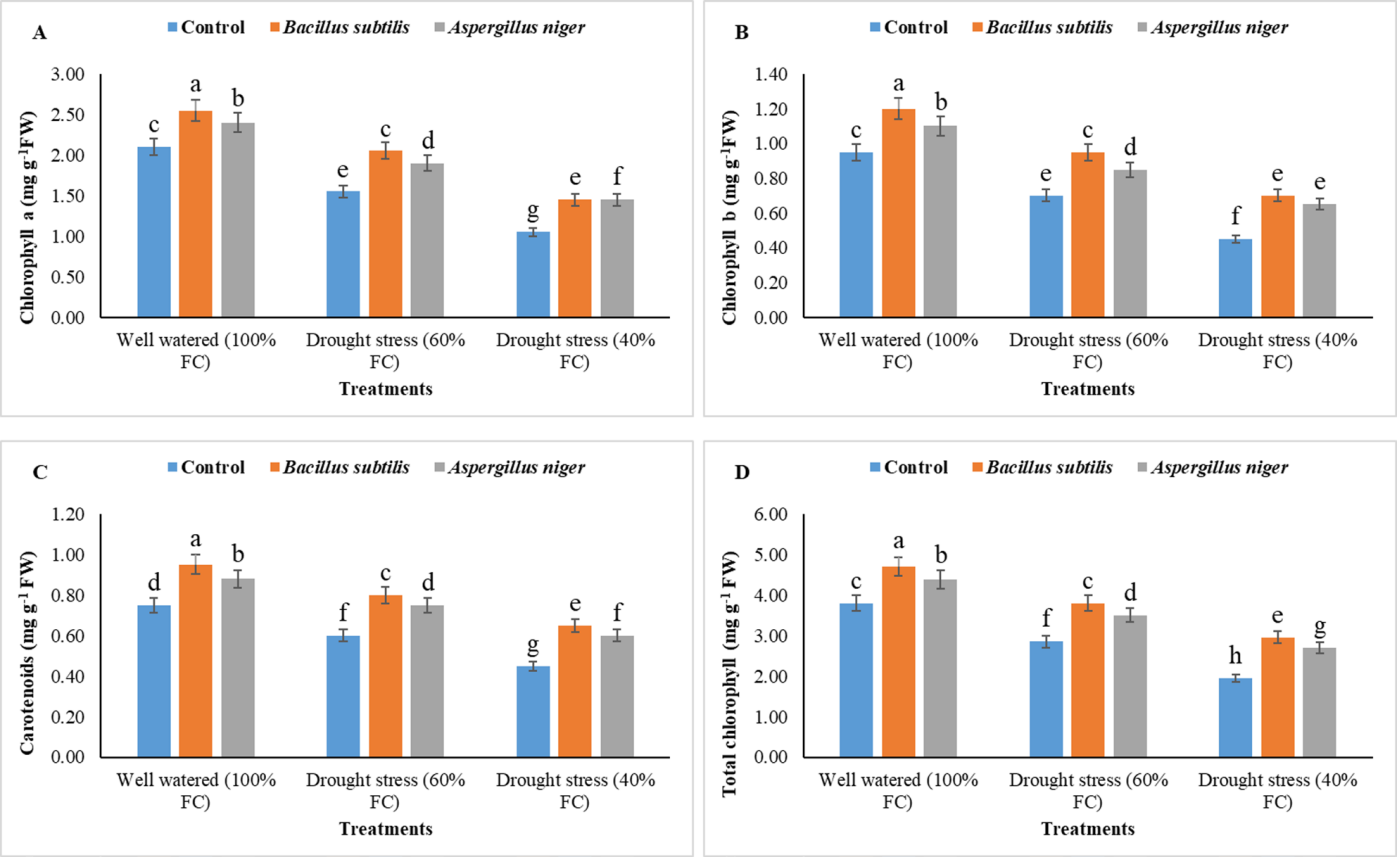
Effect of *B. subtilis* and *A. niger* on chlorophyll a (**A**), chlorophyll b (**B**), carotenoids (**C**), and total chlorophyll (**D**) content in common bean plants under drought stress. The values represent the means of three replicates with standard deviation (± SD). Mean values in each bar followed by a different lowercase letter are significantly different according to Tukey’s HSD test at *p* ≤ 0.05

Drought stress significantly reduced photosynthetic pigment contents in common bean plants, with the greatest decline observed under severe drought conditions (40% FC). In contrast, inoculation with *B. subtilis* and *(A) niger* improved chlorophyll and carotenoid contents under both normal and drought-stress conditions (Fig. 2). Under well-watered conditions (100% FC), *(B) subtilis* treatment recorded the highest pigment contents, including chlorophyll a (2.55 mg $g^{-1}$ FW), chlorophyll b (1.20 mg $g^{-1}$ FW), carotenoids (0.95 mg $g^{-1}$ FW), and total chlorophyll (4.70 mg $g^{-1}$ FW), compared with the uninoculated control. *(A) niger* also enhanced pigment accumulation, although its effect was lower than that of *(B) subtilis*.

Moderate drought stress (60% FC) led to significant decreases in chlorophyll a, chlorophyll b, carotenoids, and total chlorophyll to 1.55, 0.70, 0.60, and 2.85 mg $g^{-1}$ FW, respectively. Nonetheless, microbial inoculation reversed such decreases. The application of *B. subtilis* enhanced the pigment content compared to that of *(A) niger* when subjected to moderate drought stress. The maximum decrease in the photosynthetic pigments was observed during severe drought stress (40% FC), at which chlorophyll a (1.05 mg $g^{-1}$ FW), chlorophyll b (0.45 mg $g^{-1}$ FW), carotenoids (0.45 mg $g^{-1}$ FW), and total chlorophyll (1.95 mg $g^{-1}$ FW) were recorded for uninoculated plants. The inoculation of PGPMs led to a significant increase in pigment content even under severe drought stress. The plants subjected to *(B) subtilis* showed increased chlorophyll and carotenoid content as compared to those of *(A) niger*. This shows that there is better protection of the photosynthetic apparatus against the adverse effects of drought stress when *(B) subtilis* is inoculated (Fig. 2).

### Effect of PGPM on RWC, MSI, and EL under drought stress

**Fig. 3:**
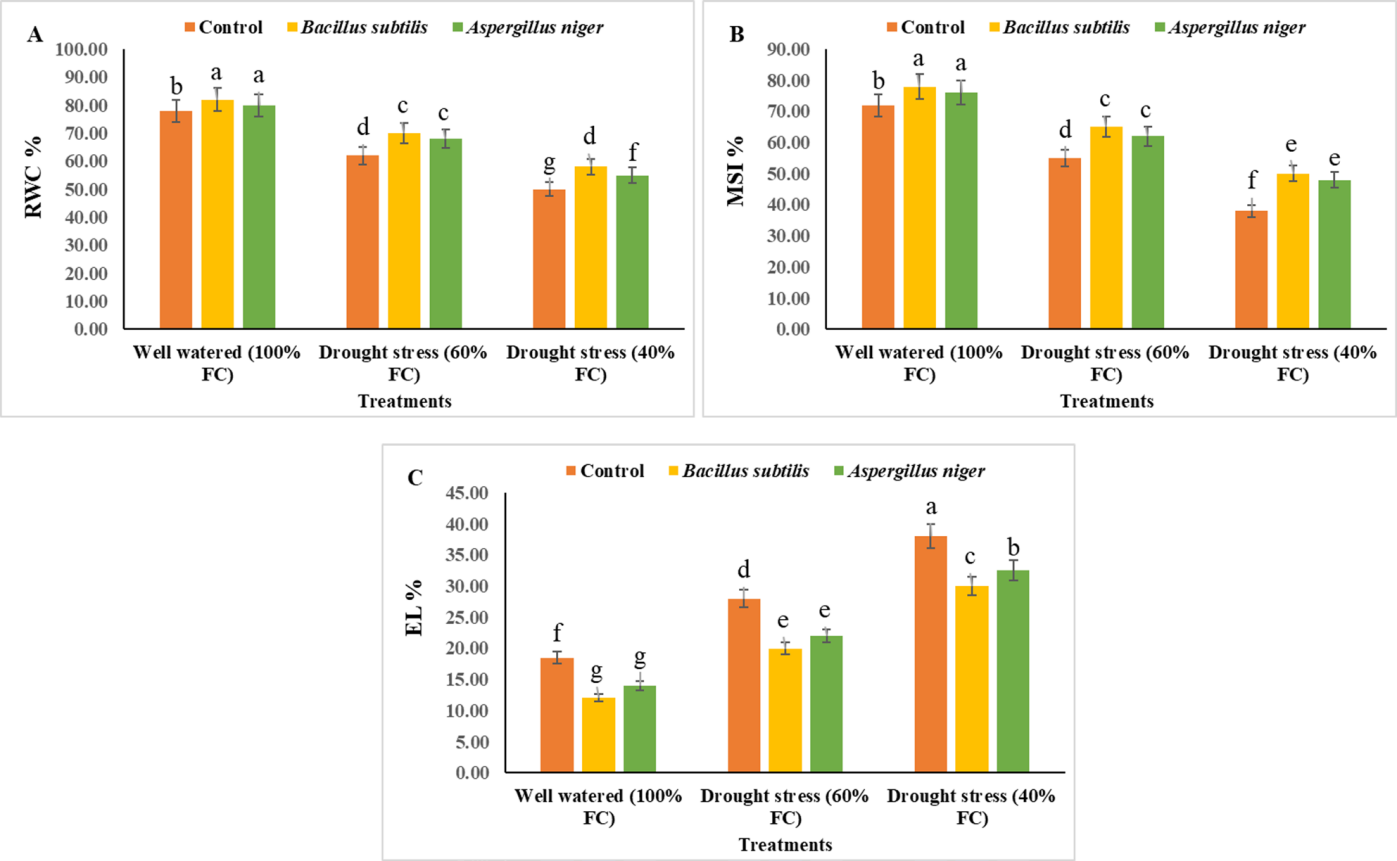
Effect of *B. subtilis* and *A. niger* on relative water content (RWC) (**A**), membrane stability index (MSI) (**B**), and electrolyte leakage (EL) (**C**) on common bean plants under drought stress. The values represent the means of three replicates with standard deviation (± SD). Mean values in each bar followed by a different lowercase letter are significantly different according to Tukey’s HSD test at *p* ≤ 0.05

Plant water status and membrane permeability were highly affected by drought stress. The RWC and MSI values decreased considerably under water deficit stress, but there was an increase in EL values. But the application of *B. subtilis* and *(A) niger* increased the water status and protected membranes from damage due to drought stress. In case of adequate water conditions (100% FC), the highest RWC (82%) and MSI (78%) were observed along with the lowest EL (12.0%) in *(B) subtilis*-treated plants compared to the uninoculated control (Fig. 3).

Drought stress at moderate intensity (60% FC) significantly reduced RWC and MSI to 62% and 55%, respectively, while EL was recorded at 28.0%. The application of PGPMs had a significant impact on improving the above-mentioned effects. Plants inoculated with *B. subtilis* maintained relatively higher RWC (70%) and MSI (65%) while having lower EL (20.5%), compared to drought-stressed plants without inoculum. Similarly, improvement was observed for plants treated with *A. niger*; however, the improvement due to *(A) niger* was comparatively low. Inoculated plants exhibited better performance for physiological parameters at severe drought stress (40% FC) compared to uninoculated plants. Under severe drought stress conditions, plants without inoculation registered the lowest RWC (50%) and MSI (38%) while having the highest EL (38.5%). Drought-stressed plants inoculated with *(B) subtilis* registered relatively higher values for RWC and MSI and relatively lower EL, at 58%, 50%, and 30.0%, respectively. Conclusively, *B. subtilis* had relatively higher performance for maintenance of cell membrane stability and water status of the host plant compared to *A. niger* (Fig. 3).

### Effect of PGPM on oxidative stress markers under drought stress

**Fig. 4:**
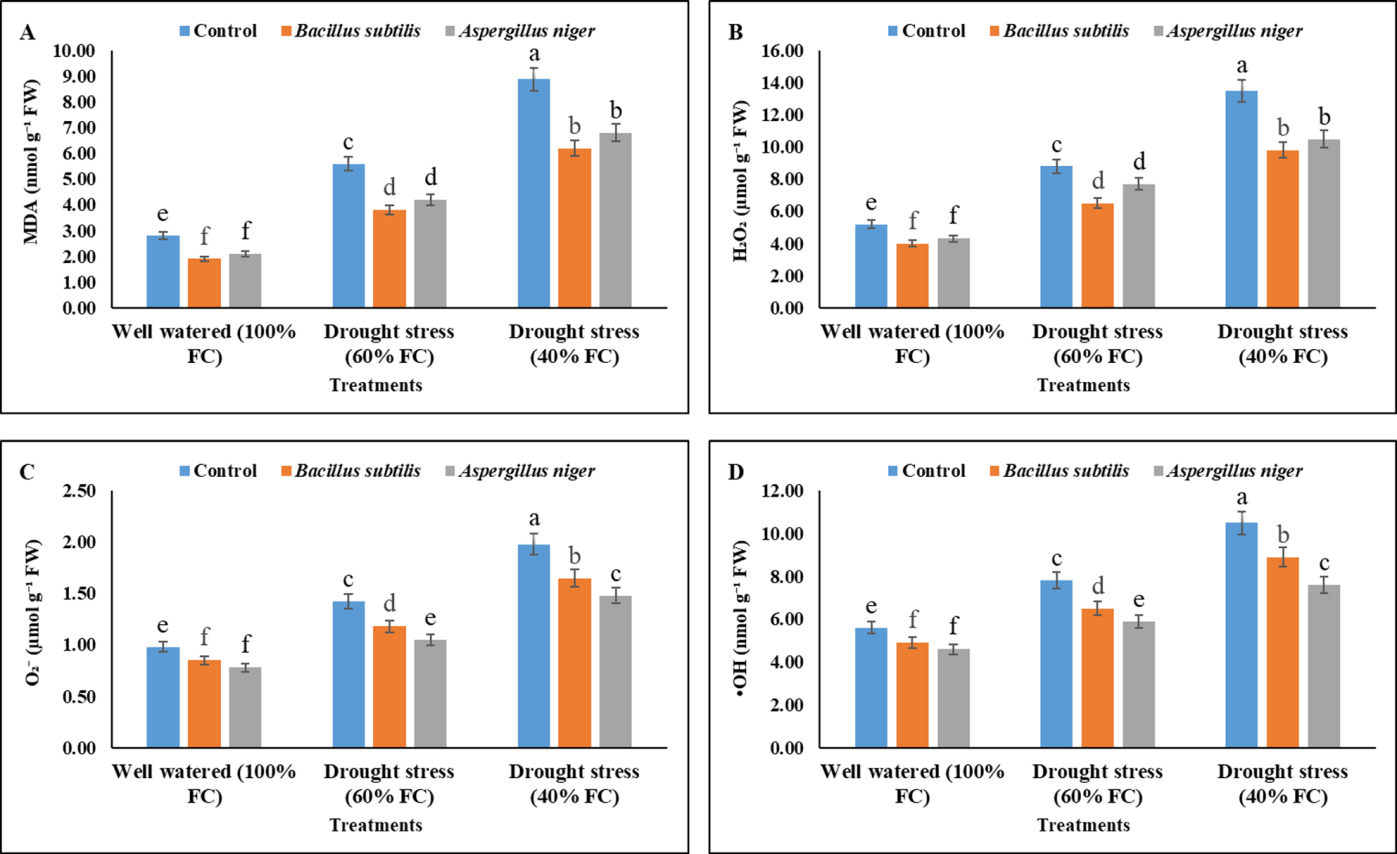
Effect of *B. subtilis* and *A. niger* on lipid peroxidation (MDA) (**A**), hydrogen peroxide (H₂O₂) (**B**), superoxide anion (O₂⁻) (**C**), and hydroxyl radicals (•OH) (**D**) on common bean plants under drought stress. The values represent the means of three replicates with standard deviation (± SD). Mean values in each bar followed by a different lowercase letter are significantly different according to Tukey’s HSD test at *p* ≤ 0.05

The occurrence of drought stress in common beans resulted in increased levels of oxidative stress biomarkers due to increased production of malondialdehyde (MDA), hydrogen peroxide (H₂O₂), superoxide radicals ($O_{2}\,-$), and hydroxyl radicals (•OH). Increased production of the above ROS was more evident under severe drought stress (40% FC). Drought tolerance induced by *B. subtilis* and *(A) niger* reduced ROS production under both normal irrigation and drought stress conditions (Fig. 4). Plants subjected to normal irrigation had lower oxidative stress biomarker levels under both inoculated and non-inoculated treatments. In the case of *(B) subtilis* treatment, the lowest levels of MDA and H₂O₂ were registered at 1.9 nmol $g^{-1}$ FW and 4.0 µmol $g^{-1}$ FW, respectively.

Moderate drought stress (60% FC) resulted in a significant increase in the levels of MDA, H₂O₂, $O_{2}\,-$, and •OH to 5.6 nmol $g^{-1}$ FW, 8.8 µmol $g^{-1}$ FW, 1.42 µmol $g^{-1}$ FW, and 7.8 nmol $g^{-1}$ FW, respectively. The application of PGPMs reduced oxidative stress, with the effect of the *B. subtilis* inoculant being better compared to that of *(A) niger*. Severe drought stress (40% FC) induced the highest oxidative stress response in the plant, whereby the level of MDA (8.9 nmol $g^{-1}$ FW), H₂O₂ (13.5 µmol $g^{-1}$ FW), $O_{2}\,-$ (1.98 µmol $g^{-1}$ FW), and •OH (10.5 nmol $g^{-1}$ FW) was higher compared to that of other treatments. PGPMs were effective in significantly lowering the oxidative markers in plants. *(B) subtilis* inoculant led to a decrease in the level of MDA and H₂O₂ to 6.2 nmol $g^{-1}$ FW and 9.8 µmol $g^{-1}$ FW, respectively. The results indicated that *B. subtilis* was better than *A. niger* in reducing the drought stress-induced oxidative stress (Fig. 4).

### Effect of PGPM on secondary metabolites and antioxidant activity (DPPH) under drought stress

**Fig. 5:**
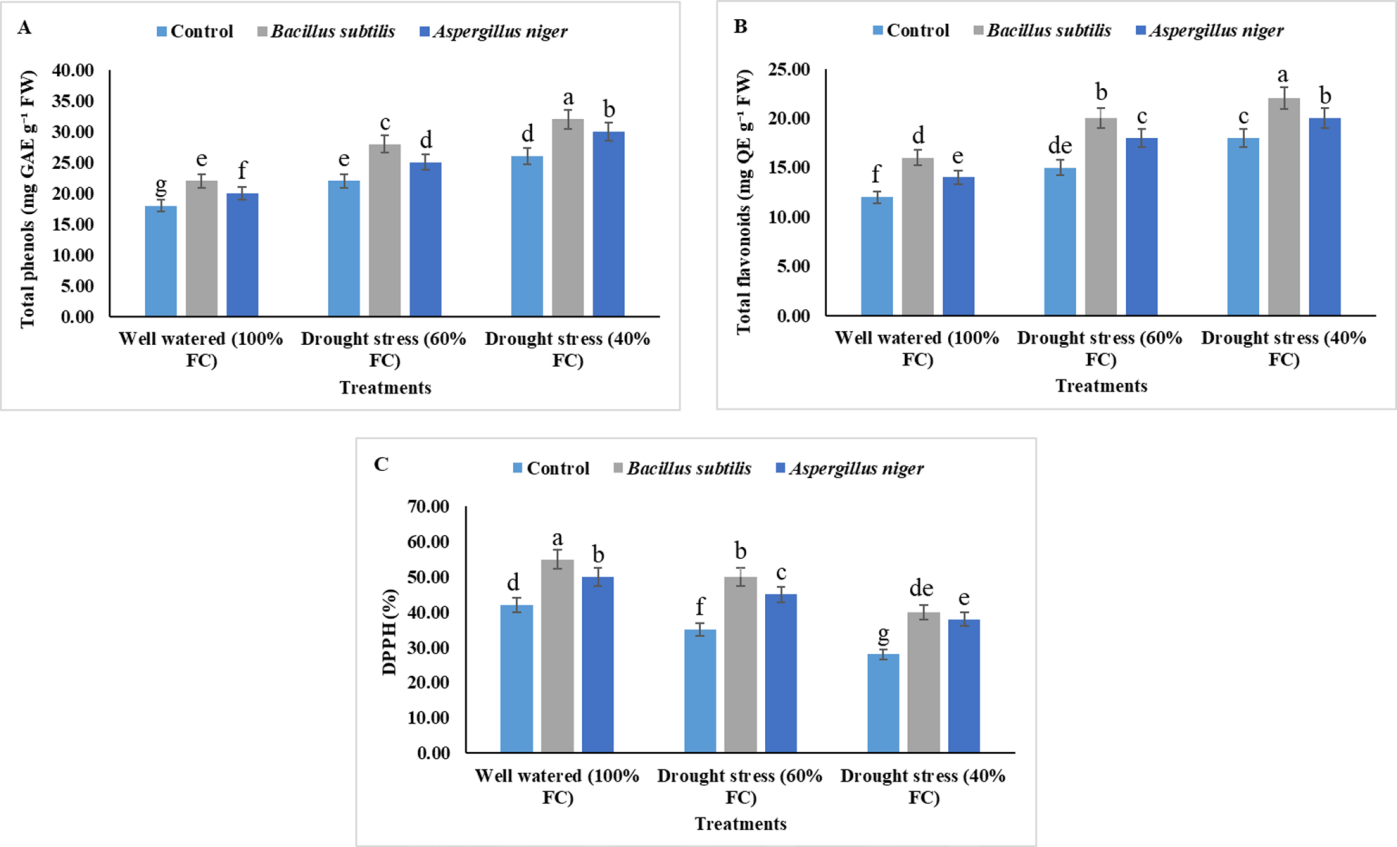
Effect of *B. subtilis* and *A. niger* on total phenols (**A**), total flavonoids (**B**), and total antioxidant activity (DPPH) (**C**) on common bean plants under drought stress. The values represent the means of three replicates with standard deviation (± SD). Mean values in each bar followed by a different lowercase letter are significantly different according to Tukey’s HSD test at *p* ≤ 0.05

Under drought stress, an increase was observed in total phenolic and flavonoid levels, whereas a decrease in DPPH radical scavenging ability was found in common bean plants, especially when subjected to severe stress (40% FC). Inoculation of *B. subtilis* and *(A) niger* not only improved antioxidant molecules but also improved antioxidant potential in both normal and drought-stressed conditions. Plants inoculated with *(B) subtilis* and *(A) niger* showed increased phenolic, flavonoid, and DPPH activities compared to control plants under 100% FC. *(B) subtilis* treatment showed maximum DPPH activity (55%), while phenolic and flavonoid contents were raised to 22.0 and 16.0 mg $g^{-1}$ FW, respectively (Fig. 5).

Under 60% FC, drought stress induced an increase in phenols and flavonoids and decreased DPPH activity to 35%. The application of PGPMs reversed these results, with *B. subtilis* inoculated plants having the highest level of phenols (28.0 mg GAE $g^{-1}$ FW), flavonoids (20.0 mg QE $g^{-1}$ FW), and DPPH activity (50%). The effect of severe drought stress (40% FC) led to the highest amount of phenolic and flavonoid content and the least DPPH activity (28%). Under drought stress, PGPM inoculation had a positive impact on the antioxidant ability of tomato plants. *B. subtilis* inoculated plants had the highest phenolic content (32.0 mg GAE $g^{-1}$ FW), flavonoids (22.0 mg QE $g^{-1}$ FW), and DPPH activity (40%), followed by *(A) niger*. In conclusion, *(B) subtilis* had a greater impact on the increase of antioxidant activity and secondary metabolites compared to *A. niger* (Fig. 5).

### Effect of PGPM on osmolyte and protein content under drought stress

Drought stress caused an increase in the level of proline, soluble sugars, and total free amino acids, along with a decrease in total soluble protein concentration in common beans (Table 3). The use of *B. subtilis* and *(A) niger* significantly improved osmolyte concentrations and protein levels during drought stress conditions. At 100% FC, inoculated plants exhibited higher amounts of proline, soluble sugars, free amino acids, and soluble proteins compared to the non-inoculated control group. Treatment with *(B) subtilis* gave the highest soluble protein level (36.0 mg $g^{-1}$ FW).

**Table 3:** Effect of *B. subtilis* and *A. niger* on osmolytes (proline, soluble sugars, and total free amino acids) and total soluble protein on common bean plants under drought stress

Treatment	Proline (µmol g⁻¹ FW)	Soluble sugars (mg g⁻¹ FW)	Total free amino acids (mg g⁻¹ FW)	Total soluble protein (mg g⁻¹ FW)
Control (100% FC)	2.5 ± 0.2^g^	12.0 ± 0.9^i^	8.0 ± 0.5^g^	28.0 ± 0.5^d^
*B. subtilis* (100% FC)	3.8 ± 0.3^f^	16.0 ± 1.0^g^	12.0 ± 0.6^e^	36.0 ± 0.9^a^
*A. niger* (100% FC)	3.2 ± 0.2^f^	14.0 ± 0.9^h^	10.0 ± 0.3^f^	33.0 ± 0.8^b^
Drought stress (60% FC)	5.8 ± 0.3^e^	18.0 ± 1.0^f^	12.0 ± 0.3^e^	22.0 ± 0.7^e^
*B. subtilis* (60% FC)	7.2 ± 0.4^d^	22.0 ± 1.1^d^	16.0 ± 0.4^c^	30.0 ± 1.0^c^
*A. niger* (60% FC)	6.5 ± 0.3^de^	20.0 ± 1.0^e^	14.0 ± 0.5^d^	28.0 ± 1.0^d^
Drought stress (40% FC)	9.5 ± 0.5^c^	24.0 ± 1.1^c^	16.0 ± 0.5^c^	15.0 ± 0.5^g^
*B. subtilis* (40% FC)	12.0 ± 0.6a	28.0 ± 1.2^a^	20.0 ± 0.4^a^	22.0 ± 0.7^e^
*A. niger* (40% FC)	11.0 ± 0.5b	26.0 ± 1.0^b^	18.0 ± 0.3^b^	20.0 ± 0.7^f^

The values represent the means of three replicates with standard deviation (± SD). Mean values in each column followed by a different lowercase letter are significantly different according to Tukey’s HSD test at *p*≤0.05

For drought-stressed plants at 60% FC, osmolytes accumulated; however, soluble protein was low, recording 22.0 mg $g^{-1}$ FW. Inoculation with PGPMs reversed the negative effects, especially for *B. subtilis*, which had higher levels of proline (7.2 µmol $g^{-1}$ FW), soluble sugars (22.0 mg $g^{-1}$ FW), amino acids (16.0 mg $g^{-1}$ FW), and soluble protein (30.0 mg $g^{-1}$ FW) compared to untreated stressed plants. At 40% FC, there was a high accumulation of proline, soluble sugars, and amino acids, but soluble protein was low at 15.0 mg $g^{-1}$ FW. There was an improvement in the levels of osmolytes and protein content after inoculating PGPMs in stressed conditions. Proline (12.0 µmol $g^{-1}$ FW), soluble sugars (28.0 mg $g^{-}$ FW), amino acids (20.0 mg $g^{-1}$ FW), and soluble protein (22.0 mg $g^{-1}$ FW) were higher in *B. subtilis* compared to *(A) niger* treatments. Overall, *(B) subtilis* was more effective than *A. niger* in enhancing osmotic adjustment and maintaining soluble protein content under drought stress (Table 3).

### Effect of PGPM on antioxidant enzyme activity under drought stress

**Fig. 6:**
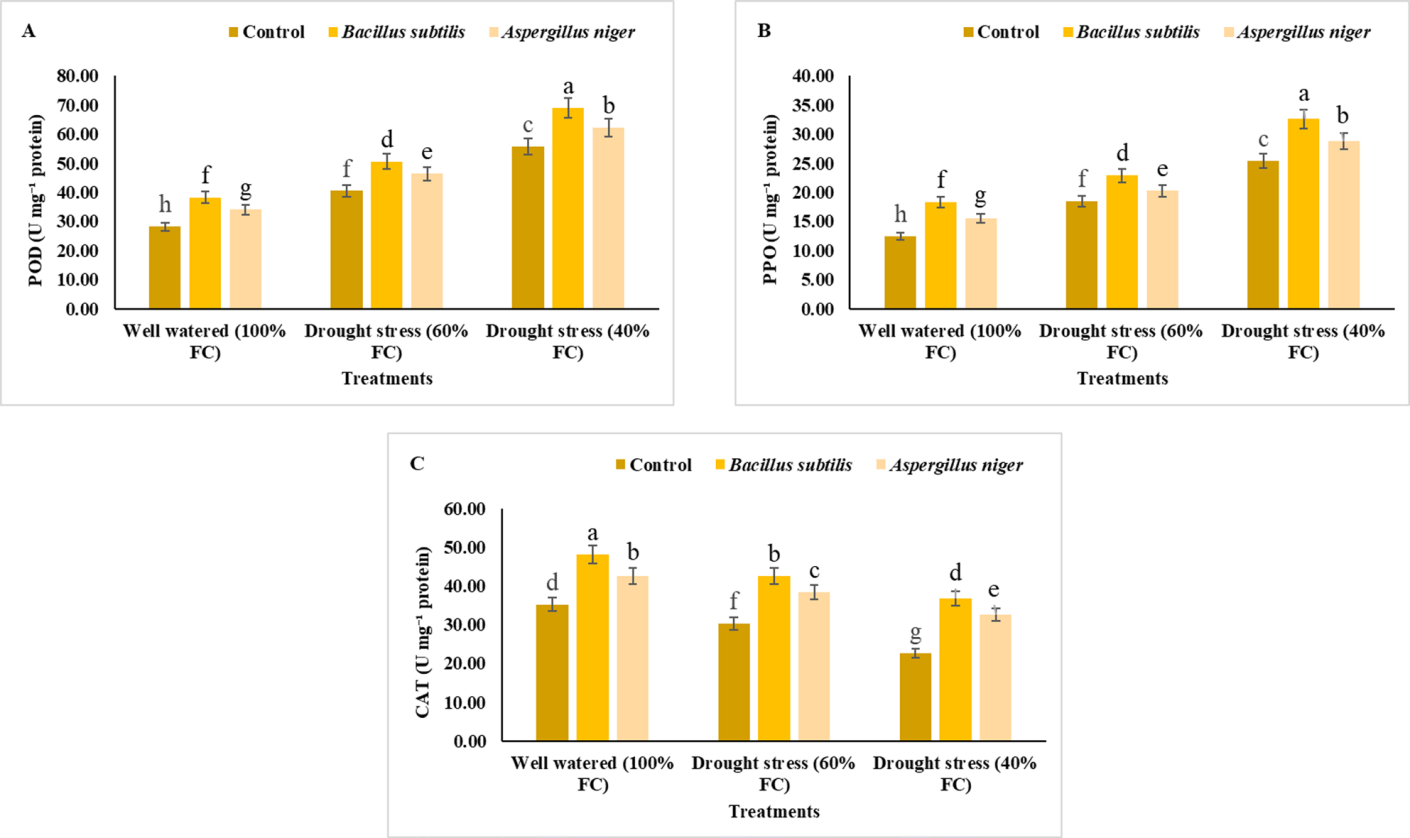
Effect of *B. subtilis* and *A. niger* on peroxidase (POD) (**A**), polyphenol oxidase (PPO) (**B**), and catalase (CAT) (**C**) activity on common bean plants under drought stress. The values represent the means of three replicates with standard deviation (± SD). Mean values in each bar followed by a different lowercase letter are significantly different according to Tukey’s HSD test at *p* ≤ 0.05

Under drought stress, POD and PPO activities were elevated, but CAT activity was decreased, especially in case of severe drought stress (40% FC). Inoculation with *B. subtilis* and *(A) niger* enhanced the activity of antioxidant enzymes and partially restored the activity of CAT under drought stress (Fig. 6). At 100% FC, the activities of POD, PPO, and CAT were higher in inoculated than non-inoculated samples, while *(B) subtilis* had the highest activity of CAT (48.1 U $mg^{-1}$ protein).

Under drought stress at 60% FC, there was an increase in POD and PPO activities, while CAT activity decreased to 30.3 U $mg^{-1}$ protein. Application of PGPM mitigated the impact of drought, where *B. subtilis* recorded elevated activities in terms of POD (50.6 U $mg^{-1}$ protein), PPO (22.9 U $mg^{-1}$ protein), and CAT (42.5 U $mg^{-1}$ protein) relative to uninoculated plants. Under drought stress at 40% FC, there was further elevation in POD and PPO activities, while CAT activity was lowered to 22.6 U $mg^{-1}$ protein. Inoculation of PGPM resulted in enhanced antioxidant enzyme activities under stress conditions. The highest levels of activities for POD (68.9 U $mg^{-1}$ protein), PPO (32.6 U $mg^{-1}$ protein), and CAT (36.8 U $mg^{-}$ protein) were recorded following *B. subtilis* treatment, followed by *(A) niger* (Fig. 6). Overall, *(B) subtilis* was more effective than *A. niger* in enhancing antioxidant enzyme activity and reducing drought-induced oxidative stress.

### Effect of PGPM on non-enzymatic antioxidant activity under drought stress

Under drought stress, there was a decrease in the content of non-enzymatic antioxidant metabolites such as ascorbic acid, tocopherol, glutathione, and anthocyanin, where the most pronounced decrease in the content of antioxidants occurred during severe drought stress (40% FC). *B. subtilis* and *(A) niger* inoculation increased the content of antioxidant metabolites in normal and drought conditions. At 100% FC, the inoculated plants had an increase in the levels of antioxidant metabolites compared to the control plant. Ascorbic acid, tocopherol, glutathione, and anthocyanin were maximum in *(B) subtilis* followed by *A. niger* (Table 4).

**Table 4:** Effect of *B. subtilis* and *A. niger* on non-enzymatic antioxidants (ascorbic acid, tocopherol, glutathione, and anthocyanin) on common bean plants under drought stress

Treatment	Ascorbic Acid (µM $g^{-1}$ FW)	Tocopherol (µM $g^{-1}$ FW)	Glutathione (µM $g^{-1}$ FW)	Anthocyanin (µM $g^{-1}$ FW)
Control (100% FC)	18.2 ± 1.1^d^	4.0 ± 0.2^d^	8.1 ± 0.4^e^	1.78 ± 0.06^e^
*B. subtilis* (100% FC)	27.3 ± 1.7^a^	5.8 ± 0.5^a^	12.4 ± 0.6^a^	2.67 ± 0.07^a^
*A. niger* (100% FC)	23.9 ± 1.1^b^	5.1 ± 0.3^b^	10.4 ± 0.5^b^	2.34 ± 0.11^b^
Drought stress (60% FC)	14.2 ± 0.8^f^	3.5 ± 0.2^f^	6.5 ± 0.1^g^	1.45 ± 0.05^f^
*B. subtilis* (60% FC)	21.6 ± 1.1^c^	5.1 ± 0.4^b^	9.8 ± 0.4^c^	2.12 ± 0.08^c^
*A. niger* (60% FC)	19.9 ± 0.9^d^	4.6 ± 0.3^c^	9.1 ± 0.5^d^	1.99 ± 0.09^d^
Drought stress (40% FC)	10.2 ± 0.6^g^	2.3 ± 0.1^g^	4.9 ± 0.1^h^	1.11 ± 0.03^g^
*B. subtilis* (40% FC)	17.0 ± 1.0^e^	4.2 ± 0.3^d^	8.1 ± 0.6^e^	1.89 ± 0.09^de^
*A. niger* (40% FC)	15.3 ± 0.8^f^	3.7 ± 0.2^e^	7.2 ± 0.5^f^	1.56 ± 0.04^f^

The values represent the means of three replicates with standard deviation (± SD). Mean values in each column followed by a different lowercase letter are significantly different according to Tukey’s HSD test at *p* ≤0.05

With 60% FC, drought stress induced a reduction in all antioxidant metabolites, with ascorbic acid and glutathione content being reduced to 14.2 µM $g^{-1}$ FW and 6.5 µM $g^{-1}$ FW, respectively. PGPM inoculation alleviated the reduction effects on these metabolites, with *B. subtilis* demonstrating increased ascorbic acid (21.6 µM $g^{-1}$ FW), tocopherol (5.1 µM $g^{-1}$ FW), glutathione (9.8 µM $g^{-1}$ FW), and anthocyanins (2.12 µM $g^{-1}$ FW) compared to uninoculated plants. At 40% FC, drought stress resulted in more significant reductions in antioxidants, with ascorbic acid and glutathione being decreased to 10.2 µM $g^{-1}$ FW and 4.9 µM $g^{-1}$ FW, respectively. Inoculation with PGPMs led to an improvement in the concentration of antioxidants. *B. subtilis* inoculation showed higher ascorbic acid (4.2 µM $g^{-1}$ FW), glutathione (8.1 µM $g^{-1}$ FW), and anthocyanins (1.89 µM $g^{-1}$ FW) than *A. niger* (Table 4).

### Heatmap analysis of traits across treatments

**Fig. 7:**
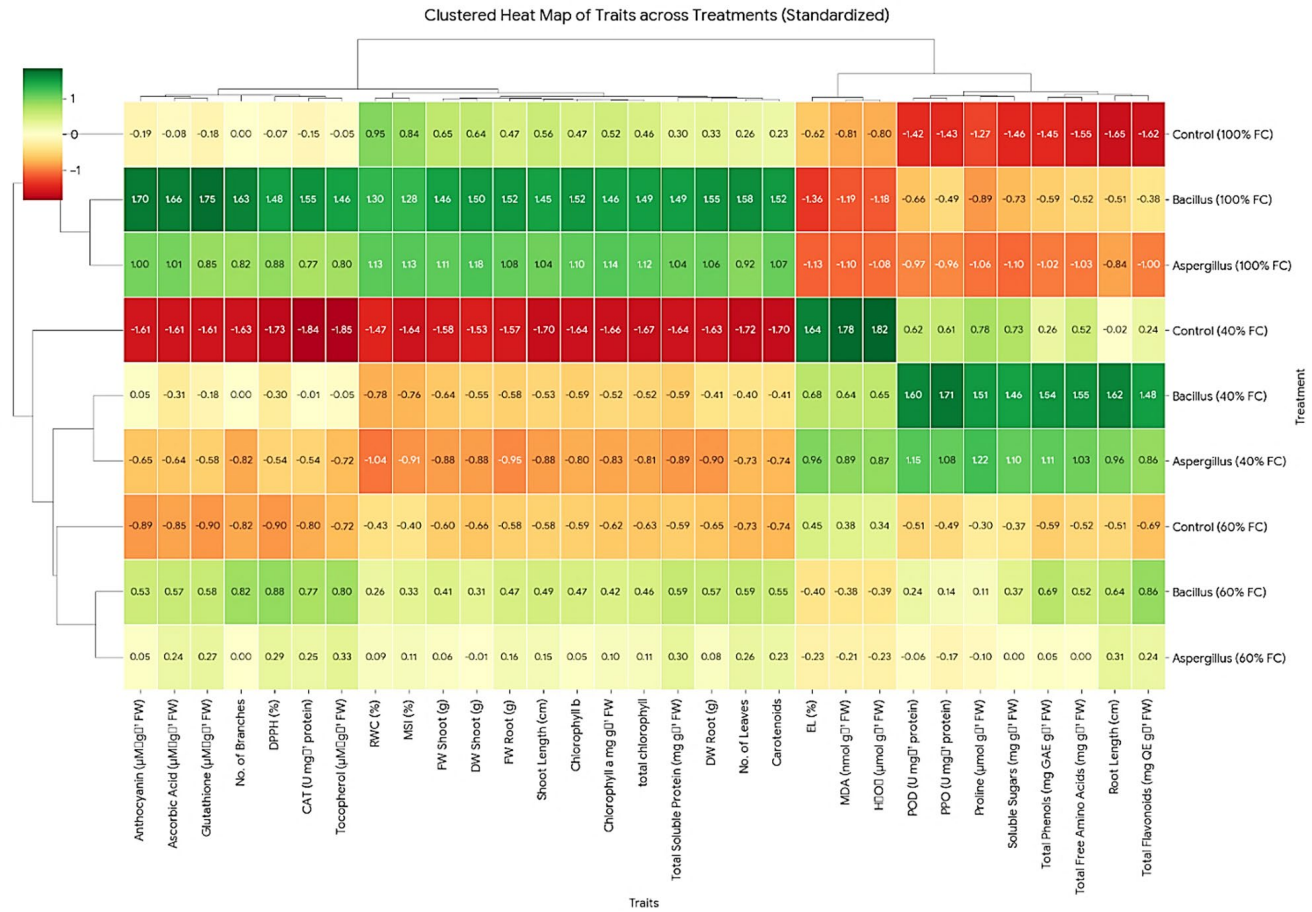
Clustered Heatmap of Biochemical, Physiological, and Antioxidant Traits under Different Water Regimes and Microbial Treatments. FW: Fresh Weight, DW: Dry Weight, RWC: Relative Water Content, MSI: Membrane Stability Index, EL: Electrolyte Leakage, MDA: Malondialdehyde, H₂O₂ (HOO): Hydrogen Peroxide, CAT: Catalase, POD: Peroxidase, PPO: Polyphenol Oxidase, DPPH: 2,2-Diphenyl-1-picrylhydrazyl (radical scavenging activity)

The clustered heatmap showed significant patterns among treatments, highlighting the impact of drought stress and microbial inoculation on plant traits. Under well-watered conditions (100% field capacity), *B. subtilis* and *(A) niger* treatments formed a distinct cluster with positive standardized values (green colors), indicating improved growth, higher photosynthetic pigments, enhanced antioxidant capacity, and better physiological status compared to the control. *(B) subtilis* (100% FC) had the strongest positive effect on plant growth (Fig. 7).

In contrast, drought stress treatments, particularly at 40% FC, clustered separately with negative values (red colors) for growth and physiological traits, reflecting significant inhibition under water stress. However, oxidative stress markers (e.g., MDA, H₂O₂) and osmolytes (like proline) were elevated, indicating stress-induced damage. Microbial inoculation improved clustering under drought conditions (60% and 40% FC), with *B. subtilis*-treated plants showing recovery in growth and antioxidant traits while reducing oxidative damage. *(A) niger* improved performance under drought but was less effective than *(B) subtilis*.

Hierarchical clustering revealed two main groups: one for favorable conditions, including the controls and *B. subtilis* and *(A) niger* at 100% FC, and another dominated by stress treatments, marked by reduced growth and heightened stress. Overall, the heatmap illustrates that drought stress negatively affects plant traits while microbial inoculation, especially with *(B) subtilis*, mitigates these effects and enhances recovery (Fig. 7).

## Discussion

The present study indicates that *B. subtilis* and *(A) niger* possess multiple plant growth-promoting (PGP) traits that collectively contribute to improved plant performance under drought stress. The production of IAA and cytokinin by *(B) subtilis* suggests a role in regulating root architecture and cellular proliferation, consistent with reports that phytohormone-producing bacteria enhance root development and nutrient uptake [52]. In addition, its ACC deaminase activity may contribute to stress modulation by reducing ethylene accumulation, thereby supporting plant growth under adverse conditions, as previously reported for *Bacillus* spp [53]. In contrast, *(A) niger* demonstrated higher phosphate solubilization potential, likely associated with organic acid secretion that converts insoluble phosphorus into plant-available forms. This aligns with earlier studies highlighting the role of phosphate-solubilizing microorganisms in improving phosphorus availability and plant growth, particularly in nutrient-limited environments [54]. Furthermore, siderophore production by *(B) subtilis* may enhance iron acquisition and indirectly suppress pathogen activity through competitive iron sequestration. Such mechanisms have been widely associated with improved nutrient uptake and plant health in siderophore-producing bacteria [55]. *B. subtilis* also showed ammonia production, which may contribute to increased nitrogen availability in the rhizosphere; however, this activity should be interpreted as nitrogen mobilization potential rather than direct nitrogen fixation. Additionally, the relatively low exopolysaccharide (EPS) production observed in both strains, particularly in *A. niger*, may still contribute to soil aggregation and moisture retention, as EPS-producing microbes are known to improve soil structure and enhance plant stress adaptation [56].

The differences observed between *B. subtilis* and *(A) niger* reflect functional complementarity in their PGP mechanisms. Previous studies have demonstrated that microbial combinations with diverse functional traits can enhance plant growth through synergistic effects on nutrient cycling and stress tolerance [57]. In this context, *(B) subtilis* appears to contribute mainly through phytohormone production and stress regulation, whereas *A. niger* primarily supports nutrient solubilization and soil improvement. Such complementary interactions support the potential use of microbial consortia as a sustainable strategy for improving crop performance under environmental stress [2, 58].

Drought stress significantly reduced plant growth, likely due to reduced water availability, cell division and elongation, impaired cell expansion, and increased oxidative stress [55–56]. These results are in accordance with Akter et al. [59], who found that water stress significantly hinders plant growth by reducing cell expansion and photosynthesis, leading to lower growth rates, biomass, and overall yield of broccoli. In contrast, inoculation with *B. subtilis* and *(A) niger* significantly increased plant growth. This improvement may be linked to their plant growth-promoting traits, including phytohormone production (IAA and cytokinin), which support cell division, root growth and development, and stress adaptation [60]. The greater response observed with *(B) subtilis* may be associated with its broader PGP activity, like phosphate solubilization ability, nitrogen fixation, and more effective plant–microbe interaction under stress conditions [61].

The photosynthetic pigments were found to be decreased due to drought stress, which suggested dysfunctional chloroplast activity and increased oxidative damage due to water shortage [62–63]. Nevertheless, inoculated plants showed relatively high levels of chlorophyll and carotenoids, implying partial safeguarding of the photosynthetic apparatus. This phenomenon could be attributed to better nutrient supply and stress tolerance due to PGPMs. There is evidence that *B. subtilis* could promote chlorophyll formation and photosystem II stability under stressful environments [64–65], whereas *A. niger* could do so via better phosphorus supply [66]. Increased carotenoid concentrations in inoculated plants could also improve photoprotection from oxidative damage [63].

The drought stress resulted in decreased RWC and MSI and increased EL, signifying that the membranes were damaged and affected the water balance of the cells. However, inoculating the plants with *B. subtilis* and *A. niger* caused an increase in RWC and MSI and a decrease in EL, which suggests that there was increased membrane stability under stress conditions. This is attributed to the production of osmolytes, water uptake by the roots, and bacterial exopolysaccharides, which may be able to increase soil aggregation and improve water retention around the plant roots, which in turn can mitigate water stress [67–69].

The effect of drought stress was associated with enhanced oxidative damage, which was shown by elevated levels of ROS, MDA, and electrolyte leakage due to restricted water supply. Drought stress results in disturbance in photosynthetic electron transfer and redox imbalance [70–73]. However, inoculating plants with *B. subtilis* and *(A) niger* showed lower oxidative stress indicators, which imply better ROS-scavenging potential. The relatively greater decrease in oxidative stress indicators after *(B) subtilis* inoculation could be attributed to higher antioxidant defense system activation [74]. This effect may be related to higher activity of antioxidant enzymes like catalase, peroxidase, and polyphenol oxidase, along with higher concentrations of non-enzymatic antioxidants like phenolic compounds, ascorbic acid, and glutathione [75–76]. As for *(A) niger*, it mainly assists through enhanced nutrient supply and stress tolerance [77–80]. Besides, decreased ROS production in plants that have been inoculated could also be attributed to increased photosynthetic efficiency and plant hydration, which reduce oxidative stress induced by drought [52, 81]. Accumulation of phenolic compounds under drought stress is considered an adaptive response, as these metabolites contribute to ROS scavenging and protection against oxidative damage [82–83]. The inoculation of PGPM, such as *(B) subtilis* and *A. niger*, induced phenolic and flavonoid accumulation under drought conditions, possibly due to the stimulation of phenylpropanoid biosynthesis pathways [80–83]. The observed phenomenon could also be attributed to the microbes’ induction of defense signaling pathways involving salicylic acid and jasmonic acid [84].

The increased scavenging ability of DPPH radicals by the plants treated with PGPM implies enhanced antioxidative potential and stability of the redox system under water deficit conditions [85]. Such a reaction could possibly occur due to higher synthesis of phenolics and proper functioning of both enzyme-based and non-enzyme-based antioxidants. The higher reaction seen in case of plants inoculated with bacteria, including elevated levels of phenolics and flavonoids, could possibly occur because of bacterial control over secondary metabolism and stress responses [86].

The occurrence of drought stress resulted in increased levels of proline, soluble sugars, and free amino acids, signifying osmotic adjustment and stress-adaptive strategies [87]. These results are in accordance with Akter et al. [59], who found that water-stressed plants showed reduced growth, yield, and leaf chlorophyll content but increased levels of ascorbic acid, proline, and dry matter of broccoli. The inoculation with microorganisms, especially *B. subtilis*, promoted an increase in osmolyte levels, which might play a role in membrane protection, ROS scavenging, and cell turgidity during stress [88]. However, the soluble protein content was found to be decreased under drought stress but increased with microbial inoculation, possibly due to improved metabolic stability and reduced protein breakdown.

Oxidative stress induced by drought stress causes disturbances in cellular homeostasis by increasing the levels of ROS, causing oxidative damage to lipids, proteins, and nucleic acids [69]. In the current study, drought caused an increase in POD and PPO activity but a decrease in CAT activity [2]. The inoculation of *B. subtilis* and *A. niger* significantly improved both enzyme-mediated and non-enzyme-mediated antioxidant systems, as seen from the increased activities of CAT, POD, and PPO enzymes and also the increased concentration of antioxidant compounds. The above-mentioned improvements might be due to the activation of plant defense mechanisms stimulated by microbes along with the increased ability to scavenge the ROS molecules [52, 89]. Higher amounts of ascorbate and glutathione indicated better performance of the ascorbate-glutathione cycle for detoxifying hydrogen peroxide radicals [90–92]. Previous studies have demonstrated that PGPR can modulate secondary metabolite profiles [93] and reduce oxidative stress in plant cells [93], which is consistent with the findings of the present study.

## Conclusion

The findings of this study suggest that plant growth-promoting microorganisms (PGPM), particularly Bacillus subtilis, have considerable potential as sustainable bio-inoculants for improving drought tolerance in common bean under water-limited conditions. The observed improvements in plant growth, physiological performance, antioxidant defense, and osmotic adjustment indicate that these microorganisms may help maintain crop productivity under drought stress while reducing the negative impacts of water deficiency. From an agricultural perspective, the use of PGPM represents an environmentally friendly and cost-effective strategy that could reduce reliance on synthetic agrochemicals and support sustainable crop management practices. In particular, *B. subtilis* demonstrated greater effectiveness than *A. niger* across most measured traits, highlighting its potential suitability for future biofertilizer or biostimulant development. However, it should be noted that the present study was conducted under controlled greenhouse pot conditions. Therefore, further investigations under open-field environments, different soil types, climatic regions, and irrigation regimes are necessary to validate the consistency, stability, scalability, and farmer-level applicability of these microbial treatments under practical agricultural production systems.

## Data Availability

All data generated or analyzed during this study are included in this published article.
